# Case report: Cutaneous metastasis of squamous cervical carcinoma: complete regression after molecular diagnosis

**DOI:** 10.3389/fimmu.2024.1528957

**Published:** 2025-01-20

**Authors:** Liwen Guo, Yanqiong Liu, Shuhua Zhang, Wei Liu

**Affiliations:** ^1^ Hunan Cancer Hospital, The Affiliated Cancer Hospital of the Xiangya School of Medicine, Central South University, Changsha, China; ^2^ Third Xiangya Hospital, Central South University, Changsha, China; ^3^ Postdoctor of Pathology of Second Xiangya Hospital, Changsha, China; Honored Professor of Pathology of Shaodong People’s Hospital, Shaodong, Hunan Province

**Keywords:** cutaneous metastasis, cervical carcinoma, prognosis, complete response, immunochemotherapy, molecular diagnosis

## Abstract

Common metastasis sites for cervical cancer are the lungs, bones, liver, brain, ovaries, and lymph nodes, among other sites. Skin metastasis is very uncommon, and is only observed in approximately 1% of patients. The cancer spreads typically through lymphatic or blood vessels, but a definitive example of lymphatic spread has not been documented thoroughly in the existing literature. Cutaneous metastasis may be confused with cellulitis or a rash; hence, an immediate cutaneous biopsy of any suspicious lesions is recommended. There is no consensus regarding the treatment of this condition. Only one documented case has shown that a combination of paclitaxel, carboplatin, bevacizumab, and zoledronic acid can lead to a complete metabolic response. Our study, which used two cycles of albumin-bound paclitaxel, cisplatin, and bevacizumab, followed by four cycles of the same regimen plus terprelimab for metastases with CPS scores of Programmed death-ligand 1 (PD-L1) over 10, resulted in a stable complete response for over eight months. Our contribution may assist in formulating effective treatment guidelines for the cutaneous metastasis of squamous cervical carcinoma in the future.

## Introduction

Cervical cancer is globally recognized as the fourth most common malignancy affecting women (1). In 2020, an estimated 604,000 women were diagnosed with the disease, and it resulted in approximately 342,000 deaths (2). The World Health Organization (WHO) launched a global strategy in 2020 to promote the elimination of cervical cancer (3). In response, the Chinese Government declared a seven-year action plan, to be implemented from 2023 to 2030, to hasten the elimination of cervical cancer, the prevalence of which has been increasing for more than 30 years (4). Cutaneous metastasis from internal malignancies is a rare phenomenon, with global incidence rates ranging from 0.1% to 1.3% (5); in China, this rate stands at 0.2% to 0.9% (6). Globally, the sites most frequently affected by primary cancer are the breasts, large intestines, lungs, skin, and ovaries (7); meanwhile, in China, the sites predominantly affected by primary cancer are the lungs, stomach, colorectum, and breasts (8). Cervical carcinoma, in particular, is associated with a higher prevalence of skin involvement, with a reported rate of 1.3% globally (5) and 0.9% in China (6). The areas commonly affected by skin metastases include the abdominal wall, vulva (as seen in our case), and the anterior chest wall (9). Uncommon sites may also be affected, such as the scalp, back, surgical drain sites, and extremities (10–13). Typically, cutaneous metastasis emerges months to years after the initial pathological diagnosis and treatment of cervical carcinoma. Most clinical diagnoses before clinical treatment are made after a pathological examination, with molecular diagnosis not performed in order to rule out primary skin carcinoma or metastasis from second squamous cell cancer in other sites. The surgical removal of the involved skin followed by radiotherapy or conservative therapy, is recommended for such patients (14). It is unsurprising that the prognosis for these patients is generally poor, often due to metastasis to other organs such as the brain, lung, and bone before or after treatment. On average, survival is around six months, with only about 20% of patients living beyond one year (2). In our report, the cancer cells surviving after radical surgery and radiochemotherapy firstly appeared in the left inguinal lymph node, and then spread to the right inguinal lymph node; they finally spread to the vulva, labia majora and minora, inguinal area, lower abdomen, the root of the right thigh, and the upper one-third of the left thigh. Nevertheless, we present the first case of cutaneous metastasis from cervical carcinoma in China where stable complete regression was achieved following comprehensive treatment based on pathological and molecular diagnoses. The patient has now remained well for over eight months.

## Clinical history

On May 27, 2019, our case involved a 52-year-old southern Chinese woman with a body weight of 72 kilograms who complained of post-coital bleeding in the past year, with the bleeding worsening within the last month. Her mother had a history of colon cancer. An examination of her pelvis upon admission showed a shallow posterior vaginal vault, a 4 cm cervix, and a nodular neoplasm on the posterior lip that bled easily upon contact; the right peripheral tissue was slightly shorter, but had moderate elasticity. No other significant physical abnormalities were observed. A cervical biopsy conducted prior to hospitalization indicated cervical intraepithelial neoplasia, grade III. The tumor was staged as IIA, and we found the cervical tumor located in the cervical uterus; the infiltration depth nearly reached the full thickness during laparotomy. Within a week, the patient underwent an extensive hysterectomy, bilateral salpingo-oophorectomy and pelvic lymph node dissection via laparotomy. A postoperative pathological examination reported moderately to poorly differentiated squamous cell carcinoma, a cervical invasion depth with a thickness greater than 2/3, no nerve invasion, carcinoma involving the upper vaginal wall, and a total tumor size of 4 cm *3 cm* 2.5 cm. No lymph nodes metastasis was observed. In addition, the bilateral para-uterine tissues, vaginal stump and adnexa were not involved.

**Figure 1:**
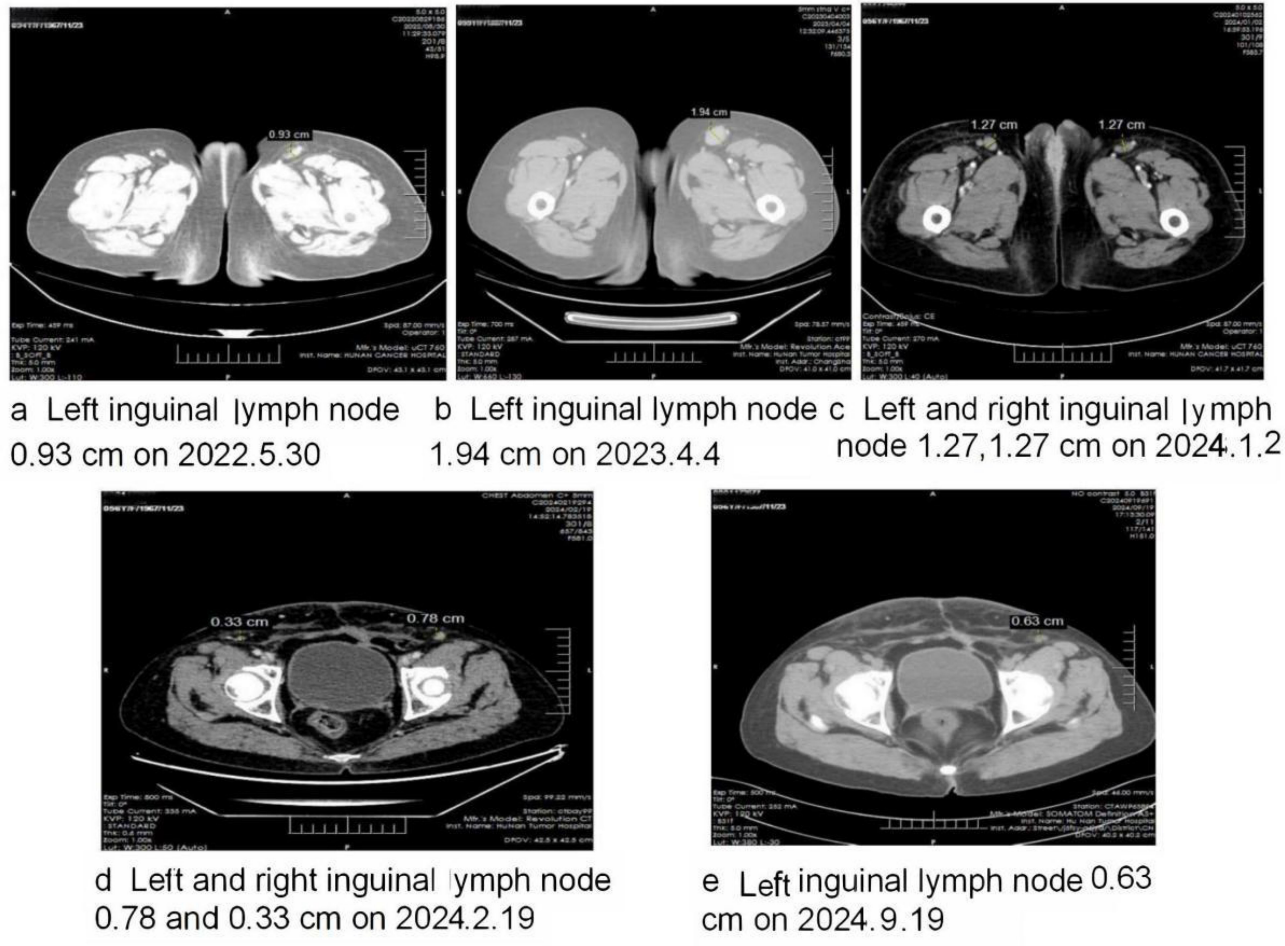
Shows the changes in the size of the dynamic inguinal lymph during the course of the disease. **(A)** Shows that the size of the left inguinal lymph node was 0.93cm on 30 May 2022, two months after the patient was enrolled in the placebo- controlled phase III clinical study. **(B)** Shows that the size of the left inguinal lymph node was 1.94cm on 4 April 2023, the day on which the clinical study was terminated; this indicated the recurrence of cervical cancer, but the patient refused in-hospital treatment. **(C)** Shows that the left inguinal lymph node was smaller but that the right inguinal lymph node was significantly larger when molecular diagnosis was used to establish the occurrence of skin metastasis from cervical cancer. **(D)** Shows that, after two cycles of albumin-bound paclitaxel, cisplatin and bevacizumab, the inguinal lymph nodes were significantly smaller. **(E)** Shows that after four cycles of albumin-bound paclitaxel, cisplatin, bevacizumab and albumin-bound paclitaxel, cisplatin, bevacizumab and terprelimab, the inguinal lymph nodes regressed to a normal size, and that the skin metastasis completely regressed.

The patient was given three cycles of docetaxel 110mg + carboplatin 500 m; 24 rounds of pelvic external radiotherapy DT4800c gy were then performed, with synchronous chemotherapy terminated due to the severe suppression of bone marrow. Then, the patient was administered docetaxel 110 mg+cis–platinum 100 mg for three cycles. Due to the significant enlargement of the left inguinal lymph node (**Figure 1A**), which is highly suggestive of recurrence, the patient was enrolled in a placebo-controlled phase III clinical study of combination immunochemotherapy (the use of AK104 730 mg or placebo + bevacizumab 1095 mg + paclitaxel 313.2 mg + carboplatin 50 mg for six cycles was initially planned). The use of bevacizumab was then terminated due to the occurrence of hematuria; after being administered AK104 alone for 1 cycle, the hematuria disappeared. The patient was later given AK 104 + bevacizumab for 11 cycles until March 6, 2023 (**Figure 1B**).

Despite the CT scan from the following month showing an increase in the size of the left inguinal lymph node, a subsequent CT scan four months later indicated that it had decreased in size. However, during this period, the right inguinal lymph node had enlarged compared to the previous examination (**Figure 1C**). On both occasions, hospitalization was recommended but declined by the patient.

**Figure 2:**
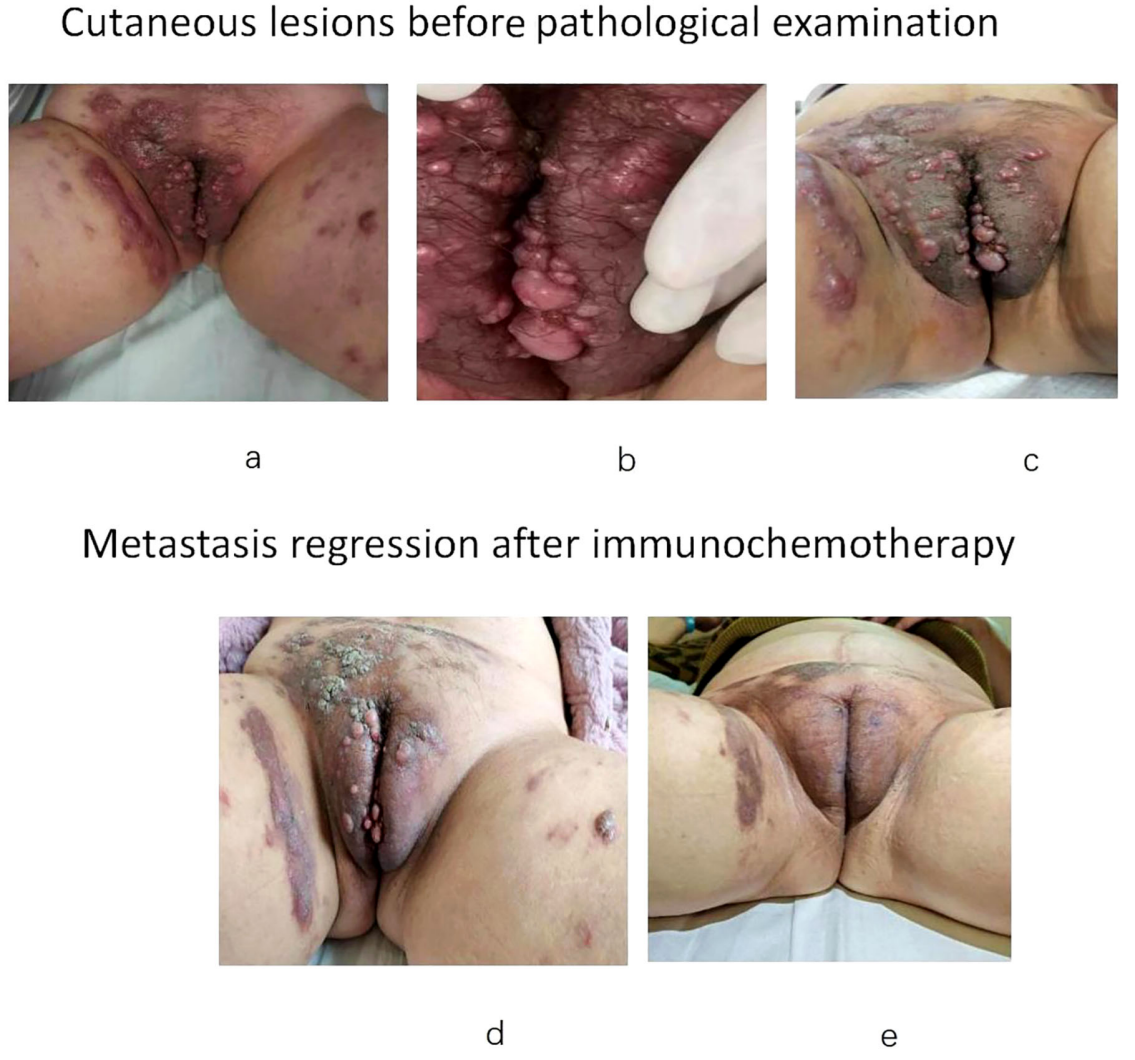
**(A)** Shows the red herpes-like solid papules and nodules of rice grain to soybean size on the pubic mound, vulva, labia majora and minora, the upper third of the left thigh, and the root of the right thigh. **(B, C)** Show the solid papules and nodules that grew to the size of soybeans or peanuts. **(D)** Shows the partial regression of skin metastases after the third cycle of changed treatment. **(E)** Shows the complete regression of skin metastasis after the fourth cycle of changed treatment, and CT confirmed the regression of inguinal lymph nodes.

**Figure 3:**
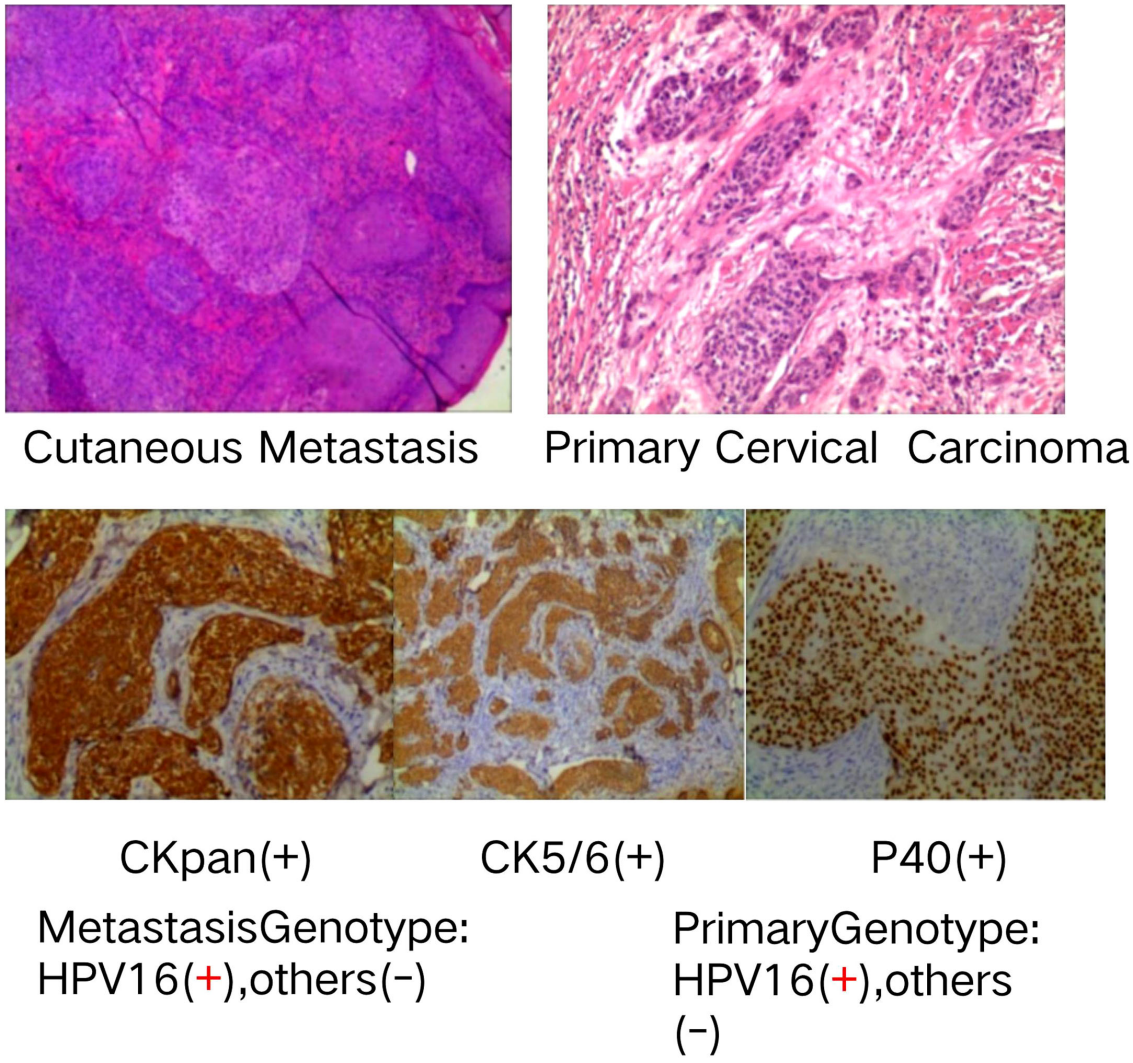
Shows the upper metastatic and moderately to lowly differentiated squamous cell carcinoma nested in the dermis, highly suggestive of the cutaneous metastasis of the primary cervical carcinoma, which was similarly differentiated and nested. The middle panel of **Figure 3** presents the immunohistochemical tests that showed the metastatic tumor cells CK(+), CK5/6(+) and P40(+), with the same pattern as primary squamous cervical carcinoma cells. The lower panel of **Figure 3** presents the primary cervical lesion and the metastatic nodule cells that show the same HPV16(+) and twenty other (-) genotypes; therefore, it is clear that these lesions are caused by the same carcinogen and exhibit the same monoclonal nature.

The patient stated that, in the second half of November in 2023, she noted red itching “herpes” and then the appearance of solid papules and nodules on the vulva that were the size of rice grains or soybeans. After 2 weeks of antiviral treatment, solid papules and nodules that were the size of soybeans or peanuts appeared on the vulva, labia majora and minora, inguinal area, lower abdomen, the root of the right thigh, and the upper one-third of the left thigh; some of these were smooth and shiny with blisters (**Figures 2A–C**). Anti-viral treatment continued to fail until December 21, 2023, and the patient was referred to the dermatology clinic of a large teaching hospital. Pathological examination revealed the appearance of metastatic and moderately to poorly differentiated squamous cell carcinoma nested in the dermis, involving the deep dermis; this was highly suggestive of the cutaneous metastasis of the previous cervical carcinoma (**Figure 3**). Subsequent immunohistochemical tests at our hospital showed the same CKpan(+), CK5/6(+), S-100 (–), P40(+), CK7(-), SOX-10(-) and SYN(-) in the tumor cells as before (**Figure 3**). HPV nucleic acid and genotype tests reported that both the cervical carcinoma and cutaneous metastasis were HPV16 (+) and other genotypes including HPV6,11,18,26,31,33,35, 39,45,51,52,53,56,58, 59,66,68,70,73 and 82(-) (**Figure 3**).

On January 2 2024, both the pathological and molecular diagnoses confirmed the post-operational cutaneous metastasis of cervical cancer. Due to the rapid development of a large vulvar mass just eight months after the last round of chemotherapy, it was evident that the patient did not respond well to the initial treatment. Consequently, the therapy was adjusted to a new regimen: albumin-bound paclitaxel 400 mg, cisplatin 125 mg, and bevacizumab 800 mg; this was administered over two cycles (**Figure 1D**). Given that the PD-L1 test for cutaneous metastasis showed a Combined Positive Score (CPS) of at least 10, in accordance with NCCN guidelines, the patient received four cycles of a modified regimen from February 19 to May 2 2024, consisting of albumin-bound paclitaxel 400 mg, cisplatin 100 mg, bevacizumab 800 mg, and terprelimab 240 mg. Notably, since March 13 2024, following the third cycle, all cutaneous and lymph node metastases had fully regressed, and the patient’s condition has remained stable and favorable (**Figures 2D, E**, **1E**).

## Discussion

Over the past five decades, the incidence of cervical cancer in the United States has reduced significantly (15). However, in China, the occurrence and mortality rates associated with cervical cancer have increased by 121.83% and 66.31%, respectively, over the last three decades (16). Fortunately, the Chinese Government has declared a seven-year action plan to accelerate the elimination of cervical cancer from 2023 to 2030. Advances in preventive measures, early detection and novel treatments could potentially curb this rising threat (17). The primary cause of cervical cancer is high-risk human papillomavirus (HPV) infection, and vaccines against HPV can prevent the onset and progression of this disease (18). Cervical cancer can spread to various organs, including the lungs, bones, liver, brain, ovaries, and lymph nodes (19).

Direct skin metastasis caused by cervical cancer is uncommon, but typically disseminates via lymphatic or blood vessels (20, 21). Considering that cervical tumor cells are often observed to obstruct lymphatic channels, it is believed that skin metastases occur due to the retrograde dissemination of the tumor as a result of lymphatic blockage (22). In our case, the interval between the initial diagnosis of cervical cancer and the appearance of cutaneous metastasis was 56 months. Upon reviewing the clinical history, this was likely due to the failure of an experimental placebo therapy that aimed to control the surviving cancer cells following surgery and chemo-radiotherapy in the left inguinal lymph node. These cells then spread to the right inguinal lymph node; thus, the left inguinal lymph node became smaller, but the patient strongly refused further hospitalization. Consequently, due to lymphatic drainage blockade, the residual cancer cells metastasized to the vulva, labia majora and minora, inguinal region, lower abdomen, the base of the right thigh, and the upper third of the left thigh. This case underscores a classic instance of cervical cancer spreading to the skin through the lymphatic system, accompanied by significant skin lymphedema (23).

The cutaneous metastasis was first misdiagnosed as a viral infection; thus, the patient was administered anti-virus treatment, but the solid papules and nodules became larger and disseminated to more areas. The biopsy of the skin nodules and pathological examination reported the cutaneous metastasis of cervical carcinoma, which was further established via the immunohistochemical results; subsequently, tests for 21 different HPV genotypes helped to identify the carcinogen, as well as to rule out primary cutaneous squamous cell carcinoma or metastasis from second squamous cell carcinoma from other sites. Our case also underscores the importance of performing a prompt cutaneous biopsy of suspicious lesions. Based on the pathological reports, molecular diagnosis and CPS of PD-L1-positive cells in the metastasis biopsy specimen, the patient was treated with two cycles of albumin-bound paclitaxel, cisplatin, and bevacizumab, followed by four cycles of the same regimen plus terprelimab. This well-tailored treatment resulted in a complete response, with the disappearance of both cutaneous and inguinal lymph node metastases. These results were consistent with similar regimens in previous reports (6, 24), and may assist in creating effective guidelines for this condition in the future. Imaging studies, including ultrasound, MRI, and PET-CT scans, confirmed no other metastases, and the patient has remained in a stable condition for over eight months. Our case also underscores the successful regimen of two cycles of albumin-bound paclitaxel, cisplatin, and bevacizumab, followed by four cycles of the same regimen plus terprelimab, which might help to create an effective guideline.

Significant advancements in the treatment of cervical cancer have been achieved in recent years, owing to enhancements in detection, refined surgical procedures, and more effective adjuvant therapies (25). Cervical cancer remains a common malignancy among women. Unlike many other cancers, cervical cancer benefits from robust prevention and screening measures. Quitting smoking, using protective methods, and receiving early HPV vaccination can lower the risk of exposure to high-risk HPV by 85%. Regular HPV testing and cervical cytology are crucial for the early detection and management of cervical abnormalities (26). For cases in which radical surgery is not immediately feasible,neoadjuvant chemotherapy can be highly effective in making locally advanced cervical cancer operable (27).

  To delve deeper into the mechanisms of this disease, we first noted that extensive hysterectomy, bilateral salpingo-oophorectomy, and pelvic lymph node dissection can be used to remove as much tumor tissue as possible. Subsequent radiochemotherapy further eliminated cancer cells that may have entered the lymphatic system, inhibiting the formation of pre-metastatic niches. In our case, this resulted in the confinement of cancer cells to the inguinal lymph nodes (28). When experimental placebo therapy proved ineffective in controlling residual cancer cells, the disease spread to both the inguinal lymph nodes and subsequently to the skin. Prompt pathological and molecular diagnosis, followed by the rapid adjustment of chemotherapy, successfully eradicated most recurrent cancer cells. The subsequent immunochemotherapy effectively eliminated the remaining cancer cells and restored anti-tumor immunity. Our findings suggest that metastasis is an active process, and that immunotherapy plays a critical role in restoring immune surveillance and response (29).

  In conclusion, for patients with cutaneous metastases following radical surgery, immunochemotherapy, guided by early skin lesion biopsy and molecular diagnosis, appears to be a promising approach.

## Data Availability

All data are available in the article or can be supplied upon request.
